# The Angular Gyrus: Multiple Functions and Multiple Subdivisions

**DOI:** 10.1177/1073858412440596

**Published:** 2013-02

**Authors:** Mohamed L. Seghier

**Affiliations:** 1 Wellcome Trust Centre for Neuroimaging, Institute of Neurology, UCL, London, UK

**Keywords:** inferior parietal lobule, connectivity, cross-modal integration, semantic, default network

## Abstract

There is considerable interest in the structural and functional properties of the angular gyrus (AG). Located in the posterior part of the inferior parietal lobule, the AG has been shown in numerous meta-analysis reviews to be consistently activated in a variety of tasks. This review discusses the involvement of the AG in semantic processing, word reading and comprehension, number processing, default mode network, memory retrieval, attention and spatial cognition, reasoning, and social cognition. This large functional neuroimaging literature depicts a major role for the AG in processing concepts rather than percepts when interfacing perception-to-recognition-to-action. More specifically, the AG emerges as a cross-modal hub where converging multisensory information is combined and integrated to comprehend and give sense to events, manipulate mental representations, solve familiar problems, and reorient attention to relevant information. In addition, this review discusses recent findings that point to the existence of multiple subdivisions in the AG. This spatial parcellation can serve as a framework for reporting AG activations with greater definition. This review also acknowledges that the role of the AG cannot comprehensibly be identified in isolation but needs to be understood in parallel with the influence from other regions. Several interesting questions that warrant further investigations are finally emphasized.

There is a rich and fascinating history of discoveries about the angular gyrus (AG). From a devastating effect on word processing when the left AG is damaged (Dejerine 1891) to triggering an out-of-body experience when the right AG is electrically stimulated (Blanke and others 2002), the AG has not yet revealed all its secrets and is still attracting a huge interest in the neuroscience community. A search on PubMed using *angular gyrus* as a keyword limited to the “title/abstract” retrieved nearly 500 studies. This brief review aims to integrate previous findings about the AG, particularly regarding its potential role(s) and whether it can be subdivided into multiple areas. The studies reviewed here are limited to functional studies of healthy populations irrespective of task or topic of interest. Given the huge number of functional neuroimaging studies that reported interesting effects in the AG, it was not possible to evaluate all their findings thoroughly. Therefore, the focus here is made on consistent results rather than the differences between previous studies. Specifically, effects of interest are limited to the functions and processes that have been shown with a compelling consistency in previous meta-analysis reviews. Because of space limitations, a large proportion of selective citations are made here of reviews with the expectation that each of these reviews would provide a comprehensive list of previous studies that reported activation in the AG in different tasks and contexts.

This review is divided into three sections. A first section provides some useful definitions about the anatomy of the AG. The second section reviews the different roles and functions that have been associated with the AG. The third section succinctly presents some of the emerging evidence of multiple subdivisions within the AG.

## The Anatomy of the AG

### Localization in the Posterior Inferior Parietal Lobule

**Figure 1.**
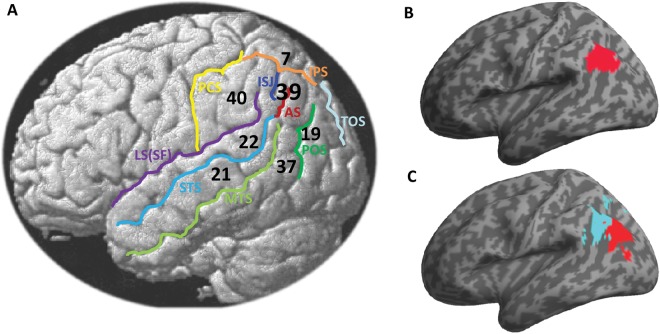
(*A*) Localization of the angular gyrus (Brodmann area [BA] 39) with respect to some anatomical landmarks that are close to the angular gyrus (AG), illustrated on a lateral view of a single subject brain. Numbers correspond to BA 7, 19, 21, 22, 37, 39, and 40. STS = superior temporal sulcus; MTS = middle temporal sulcus; IPS = intraparietal sulcus; LS(SF) = lateral sulcus (sylvian fissure); PCS = postcentral sulcus; POS = parieto-occipital sulcus; TOS = transverse occipital sulcus; AS = angular sulcus; ISJ = intermediate sulcus of Jensen. Projection of the AG from two widely used anatomical toolboxes in region-based analyses in functional neuroimaging, including (*B*) the AAL atlas (http://www.cyceron.fr/web/aal_anatomical_automatic_labeling.html) and (*C*) the probabilistic anatomy toolbox (http://www.fz-juelich.de/inm/inm-1/EN/Home/home_node.html) showing two cytoarchitectonic areas PGa (cyan) and PGp (red), as detailed in Caspers and others (2008).

The AG occupies a posterior part of the inferior parietal lobule corresponding to Brodmann area (BA) 39, von Economo and Koskinas area PG, or area 69 in the unified nomenclature of Triarhou (2007). It can be seen as the continuation of the superior/middle temporal gyri into the inferior parietal lobule with a medial boundary defined by the intraparietal sulcus (Rademacher and others 1992). Its anterior boundary with the supramarginal gyrus is marked by the descending portion of the intermediate sulcus of Jensen (Ribas 2010), and its posterior boundary is set by the dorsal part of the anterior occipital sulcus (Rademacher and others 1992). On a sagittal view, it can easily be identified by its horseshoe shape near the dorsal-posterior segment of the superior temporal sulcus that is also called the angular sulcus (Naidich and others 1995) (see Fig. 1). Recent cytoarchitectonic studies (e.g., Caspers and others 2008; Caspers and others 2006) have suggested that the AG extends rostrally to area PGa and caudally to area PGp, as illustrated in Figure 1C. Its cortical volume is estimated at around 13.2 cm^3^ and 11.7 cm^3^ in the left and right hemisphere, respectively (Rademacher and others 1992), suggesting a relative left-greater-than-right structural asymmetry (see also Eidelberg and Galaburda 1984). It is worth noting that the AG belongs to a set of parietal regions that are lightly myelinated compared with sensory or modality-specific regions (Box 1). Last but not least, an increasing literature points to an extensive training-induced structural plasticity in bilateral AG in the adult brain, particularly when learning new skills that require spatial coordination and verbal memory (see summary in Box 2).

Box 1. **AG Maturation**

Myelin content and maturational curves constitute some of the key anatomical properties that shape regional parcellation and function. In the inferior parietal lobule, it has been shown that gyration starts by 27 weeks post–gestational age, and the cortical folding of intraparietal and parieto-occipital sulci that anatomically delineate the AG may progress from 27 to 31 weeks gestational age (Dubois and others 2008). This early morphometric development may explain why the AG has been activated in previous studies in very young subjects; for instance, three-month-old infants showed strong fMRI activations in the left AG for normal mother’s voice speech compared with reversed speech (Dehaene-Lambertz and others 2002). Early studies (as reviewed in Geschwind 1965) suggested very late myelination in bilateral AG (e.g., Flechsig’s myelination map; see Figure 14 in Glasser and Van Essen 2011), being probably the latest in the whole parietal lobe. However, recent studies have observed earlier myelination than initially thought. For instance, across childhood, gray matter maturation in the AG peaks between 8.5 (Gogtay and others 2004) and 13 years (Westlye and others 2010) and then declines with age. The topology of the AG in terms of anatomical parcellation and boundaries is already well established in school-age children (Barnes and others 2011). In adults, it has been shown that the whole inferior parietal cortex, including the AG, is lightly myelinated compared with primary and secondary sensory regions (Glasser and Van Essen 2011).

Box 2. **Structural Plasticity**

There is increasing evidence about a training-induced neuroanatomic plasticity even in the adult brain that results in detectable structural changes following learning new skills (for review, see Draganski and May 2008). These structural changes can occur at the macroscopic level and thus can be revealed by morphometry analyses on high-resolution anatomical scans. Interestingly, several studies have reported structural changes in the AG during learning. For instance, when brain scans were compared in subjects before and after training to juggle, increased gray matter density was detected in the left AG and bilateral mid-temporal regions (Draganski and others 2004), probably reflecting an increase in the coordination and storage of complex visual motion. In the language domain, a comparison between bilinguals and monolinguals revealed stronger gray matter density in the bilingual brain in the anterior AG that also correlated significantly with the second-language proficiency (Mechelli and others 2004). In addition, gray matter density in bilateral AG was shown to be significantly higher in subjects who learned to read as adults (late literates) compared with a matched group of adult illiterates (Carreiras and others 2009). Recently, by measuring creative productivity scores across different skills that included visual arts, music, creative writing, scientific discovery, and invention, a whole-brain correlation between cortical thickness and creativity scores was only significant in the AG (Jung and others 2010). This literature points to a phenomenal structural plasticity in the AG when subjects are learning new skills that tap on spatial coordination, verbal storage, and creativity.

### Structural Connectivity

**Figure 2.**
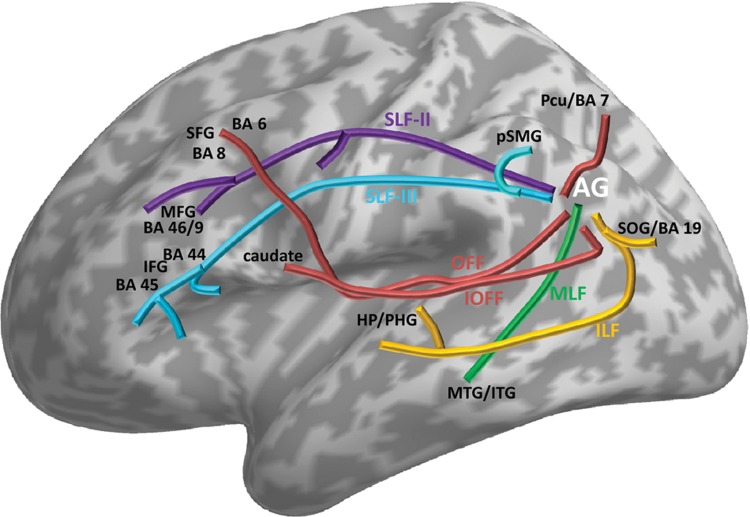
Schematic illustration of some white matter tracts mapped in previous studies that used diffusion tensor imaging and tractography analysis. It is, however, important to keep in mind some of the limitations in using such techniques to quantify structural connectivity in vivo (see review in Jones 2010; Jones and Cercignani 2010). For ease of illustration, the tracts are oversimplified (i.e., not shown at their exact extent and localization). The names of the tracts are kept identical to the ones used in the original studies. The exact localization and full extent of each tract can be retrieved from their original studies as explicitly listed below: SLF-II = superior longitudinal fasciculus–second branch (cf. Figure 3 of Makris and others 2005); SLF-III = superior longitudinal fasciculus–third branch (cf. Figure 8 of Frey and others 2008); MLF = middle longitudinal fasciculus (cf. Figures 5–7 of Makris and others 2009, Figure 7 of Frey and others 2008); ILF = the inferior longitudinal fascicle (cf. Figure 5 of Rushworth and others 2006); OFF = occipitofrontal fascicle (cf. Figure 8 of Makris and others 2007); IOFF = inferior occipitofrontal fascicle (cf. Figure 5 of Uddin and others 2010). OFF and IOFF may correspond to the same tract (see discussion in Makris and others 2007), although other studies have suggested the existence of both inferior and superior OFF in humans. BA = Brodmann area; AG = angular gyrus; SOG = superior occipital gyrus; Pcu = precuneus; pSMG = posterior supramarginal gyrus; MTG/ITG = middle temporal gyrus/inferior temporal gyrus; HP/PHG = hippocampus/parahippocampal gyrus; IFG = inferior frontal gyrus; MFG = middle frontal gyrus; SFG = superior frontal gyrus.

With its location at the junction between the occipital, temporal, and parietal lobes, the AG is considered an important interface that conveys and integrates information between different modalities and processing subsystems. For instance, large-scale connectivity analyses have shown that the AG is one of the major connector hubs linking different subsystems (Hagmann and others 2008; Tomasi and Volkow 2011). It is thus essential to identify the set of regions that anatomically connect to the AG as this would shed some light on its role during different cognitive processes. Because of the lack of consensus about the exact homology of the AG between humans and monkeys, it was not possible to discuss here the rich literature of anatomical tracer studies in the nonhuman primate (see Box 3). Thanks to recent advances in diffusion tensor imaging and tractography techniques, noninvasive visualization of white matter tracts that link the AG to other brain structures has been made possible (e.g., Catani and Thiebaut de Schotten 2008; Oishi and others 2008; Wakana and others 2004). Below is a list of some major tracts and bundles from previous diffusion tensor imaging studies that defined the AG as a seed, a target, or an intermediate connecting region in their tractography analyses. These different tracts are mentioned here using the same labels as in the original studies and are schematically illustrated in Figure 2.

Box 3. **Homology with the Nonhuman Primate Brain**

An interesting observation from previous comparative studies is the striking differences in location and size of the inferior parietal cortex between humans and animals (Hyvarinen 1982; Orban and others 2006). For instance, previous reports have suggested that there is no clear homologue in monkeys to the human AG (Geschwind 1965; Zilles and Palomero-Gallagher 2001; see illustration in Figure 1 of Culham and Kanwisher 2001). This is largely because expansion of the posterior parietal lobe in humans has taken place largely in the inferior parietal lobule and in particular in the AG (Hyvarinen 1982). A few studies, however, have tentatively proposed a homology between the AG and areas 7a/PG of the macaque brain (McCulloch 1944; Petrides and Pandya 2009). Orban and others (2004) argued that the intraparietal sulcus in monkeys may correspond not only to the intraparietal sulcus in humans but also to a part of the AG (cf. Figure 2 of Orban and others 2004). Furthermore, anatomical tracer studies in the monkey have revealed rich efferent and afferent connections between the different subareas of the posterior parietal cortex with multiple inferior frontal, dorsal prefrontal, parahippocampal, hippocampal, and thalamic, caudate, cingulate, superior temporal, and frontal eye field (e.g., Andersen and others 1990; Cavada and Goldman-Rakic 1989; Pandya and Seltzer 1982; Petrides and Pandya 2009). However, because a homology between the human AG and its counterpart in the nonhuman primate is still a matter of debate, it is unclear to what extent this rich literature of monkey anatomical tracer studies can be compared with connectivity studies of the human brain (for a similar rationale, see Uddin and others 2010).

Left and right AG are interconnected via the dorsal areas of the splenium and isthmus of the corpus callosum (Park and others 2008). The AG connects to the ipsilateral frontal and opercular areas via the superior longitudinal fasciculus (SLF) (Makris and others 2005). Specifically, the second branch of the SLF (SLF-II), located at the central core of the white matter above the insula, connects the AG to the caudal-lateral prefrontal regions (Makris and others 2005), and the third branch (SLF-III) links the AG directly to the inferior frontal gyrus (Broca area) at the level of areas BA 44 (Frey and others 2008) and BA 45 (Kelly and others 2010). It is also connected to the caudal posterior temporal regions via the middle longitudinal fasciculus (Frey and others 2008; Makris and others 2009) and to other temporal regions via the posterior segment of the arcuate fasciculus (Catani and others 2005). The AG also connects to the precuneus (BA 7) and the superior frontal gyrus (BA 8) via the occipitofrontal fasciculus (Makris and others 2007), to the caudate via the inferior occipitofrontal fasciculus (Uddin and others 2010), and to both parahippocampal gyrus (Rushworth and others 2006) and hippocampus (Uddin and others 2010) via the inferior longitudinal fascicle. Regarding its connections within the inferior parietal lobule, the AG connects to a posterior part of the supramarginal gyrus via local arcuate (U-shaped) connections (Lee and others 2007). Last but not least, it is argued that the AG receives little or no direct input from primary sensory areas (see discussion in Binder and others 2009). This rich anatomical connectivity pattern (Figure 2) enables considerable interactivity between the AG and temporofrontal subsystems in addition to other medial regions such as the hippocampus, caudate, and precuneus. As detailed below, connectivity may vary with the AG subregion used in seed-based tractography analyses (see examples in Mars and others 2011; Uddin and others 2010).

## Multiple Functions

Functionally, the AG is commonly considered part of the heteromodal parietal association cortex (Rademacher and others 1992). At the system level, it is one of the major connecting hubs, as shown in previous functional connectivity studies (e.g., Tomasi and Volkow 2011). An abundant literature on healthy adult populations revealed that both left and right AG are implicated in numerous tasks and processes. For instance, using the NeuroSythn database (Yarkoni and others 2011), a “reverse inference” at coordinates [*x* = −48, *y* = −56, *z* = 36] revealed that several concepts and keywords previously have been associated with the left AG in numerous fMRI and PET studies, with the top 10 concepts including retrieval, default mode network, memory, semantic, sentence, semantic memory, consciousness, narrative, intentional, and familiar. This section succinctly reviews some of the most common functions associated with the AG on the basis that these functions consistently implicated the AG across numerous studies, as demonstrated from previous meta-analysis reviews. As a selection criterion, the AG should at least be listed in the meta-analysis tables of previous reviews. Each function is briefly introduced in separate paragraphs below. Table 1 provides some of the most consistent coordinates for the AG in different tasks from previous meta-analysis reviews.

**Table 1.** Meta-Analysis Studies That Identified Consistent Angular Gyrus (AG) Activation across Numerous Functional Neuroimaging Studies (Listed Alphabetically: First Author + Year)

	Task/Process	Left AG	Right AG
Arsalidou 2011	Visuospatial facts retrieval in calculations	—	30 –58 32^a^
Dehaene 2003	Verbal retrieval of numbers	−41 −66 36	—
Kim 2010	Memory retrieval: self-referential processing	−40 −74 24	—
	Memory retrieval: control processing	—	42 −60 26
Laird 2009	Default mode network	−42 −66 18	46 −66 16
Mar 2011	Story-based theory-of-mind	−42 −72 34	54 −54 26
	Non-story-based theory-of-mind	−46 −62 32	52 −54 34
Mazoyer 2001	Default mode network	−52 −74 18	46 –68 26
Nee 2007	Conflict resolution in go/no-go tasks	—	42 −64 32
Shulman 1997	Default mode network	−45 −67 36	—
Spaniol 2009	Episodic memory: retrieval success	−34 −60 44^a^	46 –50 28
	Episodic memory: subjective recollection	−40 −72 32	52 −68 16
Sperduti 2011	External-agency attribution	−50 −56 44	58 x02212;56 36
Spreng 2009	Default mode network	−43 –69 32	49 −63 20
	Autobiographical memory	−47 −61 26	49 −59 27
	Visuospatial navigation	−34 −78 34	42 −74 32
	Theory-of-mind	−43 −68 39	—
	Prospection	−49 –64 29	—
Turkeltaub 2010	Phoneme discrimination	−38 −60 42^a^	—
Vigneau 2006	Semantic processing	−45 −68 26	—
Vilberg 2008	Memory retrieval: successful recollection	−43 −66 38	53 −61 25
Wager 2005	Inhibition in go/no-go tasks	—	41 −64 35
Wang 2010	Concrete versus abstract concepts	−34 −76 34	—

— = AG not identified.

a The coordinates of these dorsal-medial clusters may extend to other parietal subareas of the intraparietal sulcus (see Caspers and others 2008).

But first it is important to keep in mind some conceptual issues and limitations on the kind of inferences that someone can make from previous literature (see Box 4 for more details). For instance, it is worth noting the discrepancy in previous studies and reviews when it comes to labeling their AG foci using sometimes different names for the same localization. The nomenclature of region thus tends to vary depending on the cognitive domain under study. For example, activation in the AG is more often described as (or part of) the *temporoparietal junction* in social cognition. In the language and reading literature, the labels *posterior middle temporal gyrus* and *temporo-parieto-occipital cortex* are sometimes used. Other studies, particularly in the domain of attention, default mode, number processing, and memory, have used unspecific labels such as *inferior parietal lobule, posterior parietal cortex*, or *ventral parietal cortex* to designate their AG activation, as these labels mix AG with other neighbor regions such as the supramarginal gyrus and the lateral bank of the intraparietal sulcus. In particular, several distinct areas may exist along the banks of the intraparietal sulcus (e.g., Culham and Kanwisher 2001; Grefkes and Fink 2005; Konen and Kastner 2008), and thus it is important to not conflate them with the AG. When these labels were used, the stereotaxic coordinates (if provided) were compared with typical localization of the AG (as defined in previous atlases of Fig. 1B,C) to decide whether to include such activations in the review.

Box 4. **Methodological Issues**

1. Identifying the exact role of a given brain structure is not straightforward in the context of the many-to-many relationship between structure and function. In this context, the exact role of the AG critically depends on the set of regions it is interacting with during a given task/process. This implies that the role of the AG cannot comprehensibly be identified in isolation but ideally needs to be understood in parallel with the influence from other regions—for instance, by combining the AG with other interacting regions to enable meta-analyses at the *system* level. This kind of systemic meta-analysis may help, for instance, to identify brain regions that are consistently coactivated with the AG and how they interact with task demands, modality, and stimulus domain.
2. Existing differences in functional properties and lateralization between the left and right AG have not always been taken into account in previous studies and reviews, although strong bilateral AG activations are not uncommon. Indeed, some interesting differences between the left and right AG may get “blurred” and thus were omitted in previous meta-analysis reviews; therefore, differences between the left and right AG are not strongly emphasized in this review.
3. It is obvious to note the diversity in tasks that were previously used to activate the AG, varying in terms of stimulus material, domain, modality, baseline condition, response type, paradigm design, and sequence. In the same way, reporting activation in the AG during a given function/domain with fMRI or PET (i.e., typically by contrasting different conditions/tasks) does not necessarily mean that the AG was essential for that function/domain. Here, emphasis was particularly put on consistent findings, and thus such inevitable methodological differences were ignored.
4. There is a sort of “borrowing” of several concepts across different domains for the interpretation of activations in the AG, which inevitably resulted in the same concept being associated with different processes in completely different tasks. For instance, the use of “magnitude/size” in spatial attention and number-processing studies, “referential” in social cognition and the default network studies, or “semantic” in language and episodic memory studies may reflect different processes despite being described under the same concept.
5. Previous studies and reviews have used different terms to describe the responses that are common (or independent) to several modalities, including *multimodal, heteromodal, trimodal, amodal, intermodal, cross-modal, polymodal, transmodal*, and *modality independent*. Although subtle differences exist between these concepts, the term *cross-modal* is used here to stress the significant activation in the AG across several modalities.

### Semantic Processing

Semantic processing is the most consistent function that activates the AG, particularly in the left hemisphere. Early functional imaging studies demonstrated left AG activation during semantic tasks on auditory (Demonet and others 1992) and visual (Vandenberghe and others 1996) stimuli, and these findings have been replicated with high consistency and reliability across numerous studies (see meta-analysis reviews in Binder and others 2009; Vigneau and others 2006). For instance, Binder and others (2009) found that the most consistent semantic activation across 120 functional neuroimaging studies was located within the left AG (Binder and others 2009), with a less strong but consistent effect in the right AG as well. Moreover, the AG plays a major role in processing concrete relative to abstract concepts (Wang and others 2010). More specifically, the left AG seems to be engaged in all aspects of semantic processing that require concept retrieval and conceptual integration (Binder and others 2009), and it provides semantic constraints during language comprehension (Price CJ 2010; Seghier and others 2010). As detailed below, semantics are inherently present in multiple tasks that activate the AG.

### Reading and Comprehension

The involvement of the AG in reading comprehension was first suggested by Dejerine (1891), who documented a loss in the capacity to read and write words following damage to the left AG. This finding was then popularized by the seminal work of Geschwind (1965). In his model, Geschwind (1965) defined the AG as a visual memory center for words that turns written language into spoken language and vice versa. Activation in the left AG during reading was found to be highly consistent in children as well (see meta-analysis in Houde and others 2010); however, the left AG was not sensitive to visual word forms because it was not activated during reading aloud single words relative to fixation (see discussion in Price CJ 2000). This nonselectivity to word forms is also supported by several meta-analysis reviews showing that the AG was not consistently activated in single word reading in skilled adult readers (Fiez and Petersen 1998; Purcell and others 2011; Turkeltaub and others 2002). As discussed below in the next paragraph, this absence of activity for words relative to fixation might be explained by the strong task-independent deactivation that is typically seen in bilateral AG. This deactivation relative to fixation and rest has been attributed to greater semantic associations during free thought than during reading (Binder and others 1999). A semantic account of the AG during reading was also supported by the strong positive correlation between activation in bilateral AG and both word frequency and imageability (Binder and others 2005; Graves and others 2010). However, Graves and others (2010) argued that the AG is not involved in mapping from semantics to phonology, a role attributed to middle and inferior temporal gyri, but it is likely to support semantic feature knowledge (Graves and others 2010). Thus, what emerges from this large literature is that the AG engages in reading when semantic associations are made (Price CJ and Mechelli 2005), an involvement that is particularly enhanced during sentence reading and more generally in comprehension of speech and written language (Obleser and Kotz 2010; Xu and others 2005). In addition to this dominant semantic account in comprehension, it is worth noting the potential role of the AG in phoneme discrimination in sublexical speech perception (see meta-analysis in Turkeltaub and Coslett 2010).

### The Default Network

The “default network” or the “default mode network” (Greicius and others 2003; Raichle and others 2001) designates a set of brain regions that are strongly deactivated during goal-directed tasks as compared with *rest* or passive baselines. It is thought to participate in internal *mentation* that becomes prominent when people are not engaged in external interactions (Buckner and others 2008), and it forms one of the most consistent resting-state networks (Smith and others 2009). These task-independent deactivations include specifically the bilateral inferior parietal, medial frontal, and posterior cingulate cortex. The deactivation in the inferior parietal cortex that includes the bilateral AG is remarkably reliable (Shehzad and others 2009) and consistent across different tasks, paradigms, subjects, and studies (see recent meta-analysis reviews in Buckner and others 2008; Laird and others 2009; Spreng and others 2009). Several hypotheses have been put forward to explain such consistent deactivations in the AG. One influential hypothesis suggests that the AG is involved in task-free semantic and conceptual processes that result from the manipulation of acquired knowledge about the world during rest that is interrupted during effortful tasks (Binder and others 1999; see also Seghier and others 2010; Wirth and others 2011). Others have suggested that the bilateral AG act as dynamic self-referential regions during rest that are associated with significant behavioral profiles in interoception and somesthesis (Laird and others 2009). Alternatively, the AG might be engaged in constructing mental scenes based on memory during rest or when subjects envision themselves in the future (Andrews-Hanna and others 2010). To summarize this paragraph, the common denominator between these different hypotheses is the engagement of the AG in the manipulation of conceptual knowledge and mental representations when the mind wanders during “rest.”

### Number Processing

Early neuroimaging studies have shown strong AG activation during digit subtraction (Roland and Friberg 1985) that has been replicated with high consistency across functional studies with varieties of tasks that manipulated different numerical operations and presentations (for review, see Dehaene and others 1998; Dehaene and others 2003). For instance, the AG has been shown to mediate spatial representations of numbers (Gobel and others 2001) and might be specific to Arabic digit perception even under passive tasks (Price GR and Ansari 2011); however, its specificity for numbers is still debatable. For example, bilateral AG were activated during a conceptual decision on numbers, but this activation was similar to conceptual decisions on object names in the left AG (Cappelletti and others 2010), which argues against a selective role of the left AG for number processing (e.g., Cappelletti and others 2007). In this context, Dehaene and others (2003) argued that the left AG is mainly involved in the verbal coding of numbers because it was strongly activated during small problems of addition and multiplication that require the retrieval of arithmetic facts stored in the verbal memory. For example, by comparing problem solving of small versus large problems over different arithmetic operations, a significant difference was found in the left AG (Grabner and others 2009), which supports its role in arithmetic fact retrieval. Interestingly, the left AG seems also to play a major role during the transfer of facts between arithmetic operations (Ischebeck and others 2009). Although the left AG has dominated the number-processing field, activations in the right AG have not been infrequent. For instance, in a recent meta-analysis, the right AG has been shown to be a highly consistent cluster that is most likely to be involved in visual-spatial attention when calculation problems are being solved (Arsalidou and Taylor 2011).

### Attention and Spatial Cognition

Several functional neuroimaging studies have suggested strong AG involvement in attention mechanisms (see review in Corbetta and Shulman 1998; Singh-Curry and Husain 2009). In particular, the AG might be involved in the reorienting or shifting of attention—for instance, when shifting the attentional system toward particular stimuli that have high salience in terms of motion, emotion, value, or meaning (Gottlieb 2007). For example, it has been shown that attentional reorienting originates in the right AG thanks to its causal role in using task history to update attentional selection (Taylor and others 2011). The right inferior parietal lobule including the AG may also play an important role in maintaining attention as well as encoding salient events in the environment (Singh-Curry and Husain 2009). Furthermore, in their recent meta-analysis about the attention and memory systems, Ciaramelli and others (2008) argued that the inferior parietal cortex, including the supramarginal gyrus and the AG, is part of a “bottom-up” attentional subsystem that mediates the automatic allocation of attention to task-relevant information (Ciaramelli and others 2008), particularly in attending to retrieved memories (Cabeza and others 2008).

It is interesting to note that the bilateral AG are involved in a wide range of tasks in spatial cognition, which reflects our ability to process and integrate all spatial aspects of our environment, including the spatial analysis of external sensory information and internal mental representations (for review, see Sack 2009). One common example of such spatial cognition processes is the ability to discriminate left from right. This left-right discrimination consists first of a perceptual or spatial encoding process and then the ability to associate each side with the word *left* or *right*. As shown recently, the left AG is the site where spatial information is integrated with the meaning of the words *left* and *right* (Hirnstein and others 2011). Interestingly, the important supporting role of the AG in spatial cognition, particularly in the right hemisphere, explains why the AG is critical for perceptual learning (see discussion in Rosenthal and others 2009). This again highlights the major role of the AG in integrating spatial information with conceptual knowledge.

### Memory Retrieval

The AG is associated with verbal working memory, particularly during the retrieval of verbal material (Jonides and others 1998). Recent meta-analysis reviews have demonstrated a strong involvement of the AG during episodic memory retrieval, particularly during successful (Ciaramelli and others 2008; Vilberg and Rugg 2008) and subjective recollection (Spaniol and others 2009). In addition, bilateral AG, as part of the inferior parietal lobule mediating the automatic “bottom-up” attentional resources (Cabeza and others 2008), play a critical role in monitoring the retrieval output continuously, with an activation level that increases when memory performance is high and when recognizing items with high compared with low confidence (Cabeza 2008; Ciaramelli and others 2008). However, others have argued that attention and memory can take distinct forms in the posterior parietal cortex, with the contribution of attention to memory varying between the left and right AG, and may differ between episodic encoding and retrieval (Hutchinson and others 2009). Moreover, AG activity has also been shown to act as a marker of violations in memory expectations, where a violation reflects a lack of correspondence between retrieval outcomes and expectations (O’Connor and others 2010). Others have alternatively suggested that the AG, particularly in the left hemisphere, serves as a memory buffer for intention maintenance that sustains episodic information until the execution of an action (e.g., Kalpouzos and others 2010). Another set of evidence is provided by studies that compared AG involvement in episodic memory with the default network (e.g., see meta-analysis in Kim 2010). For instance, Sestieri and others (2011) compared the activation profile of the default network during episodic memory retrieval and found that the strongest memory search–related activations were observed in the bilateral AG (Sestieri and others 2011). Last but not least, the AG is also part of the autobiographical memory system (Spreng and others 2009; Svoboda and others 2006). It is worth noting that the important role of the AG in memory is facilitated by its strong connectivity with the hippocampal system as mentioned above (i.e., Fig. 2).

### Conflict Resolution

This concerns a broad range of tasks where participants need to select or execute an appropriate response in the context of conflict or interference from other conditions or stimuli. This conflict can be semantic, spatial, or emotional in nature (see examples in Fan and others 2003). One particular class of conflict tasks that strongly activated the AG are go/no-go tasks that present participants with two types of stimuli, one requiring a response and the other requiring the withholding of a response. Specifically, the right AG was found to be strongly involved during the inhibition of the inappropriate response across a variety of go/no-go tasks (see meta-analysis reviews in Nee and others 2007; Wager and others 2005). Regarding the left AG, previous meta-analysis reviews failed to identify consistent activations across neuroimaging studies that used different conflict tasks. One possible hypothesis is the need for a strong contextual/semantic conflict to activate the left AG. For instance, when comparing between three different conflict tasks, the right AG was strongly activated in all conflict tasks, whereas the left AG was only involved in a sentence comprehension task that included a conflict between plausible and implausible sentential representations (Ye and Zhou 2009). In the same way, when manipulating semantic and referential anomalies during sentence comprehension, the right and left AG were strongly involved in solving referential ambiguity (Nieuwland and others 2007).

### Theory-of-Mind and Social Cognition

Theory-of-mind or mentalizing is a framework used in social cognition to infer the mental states of others (i.e., the attribution of mental states) at the level of their beliefs, emotions, goals, and motivations. It is an essential capacity that helps the human brain to *reason* about other people to effectively communicate and navigate in the social world. Theory-of-mind tasks can be verbal or nonverbal in nature and typically involve false belief stories, attribution of mental state to one or more characters of a story, cartoon stories, or animations of rigid geometric shapes that depict social interactions (e.g., Gallagher and others 2000). In this large literature of social cognition, a consistently activated region extended over posterior temporal to posterior parietal cortices (Decety and Lamm 2007), including the AG (Mar 2011). For instance, numerous functional neuroimaging studies have shown strong involvement of bilateral AG in theory-of-mind or mentalizing tasks (see meta-analysis reviews in Buckner and others 2008; Mar 2011; Spreng and others 2009). As shown recently, bilateral AG showed strong involvement in both (verbal) story-based and (nonverbal) non-story-based theory-of-mind tasks, which may suggest its involvement in some aspects of sequencing and scene construction (see discussion in Mar 2011). Alternatively, other studies have suggested a role of bilateral AG in theory-of-mind in accessing story content and episodic memories (Calarge and others 2003), in external-agency attribution (Sperduti and others 2011), or when inferences about human intention are made during discourse processing (Mason and Just 2011). In sum, the AG in social cognition seems to support access to mental representations and judgment making on contextual associations.

### AG as a Cross-Modal Integrative Hub

It is striking to see the high similarity in task-free deactivation in the AG as in the default network with multiple networks that, in addition to the ones cited above, also included other high-order (meta-cognitive) systems such as envisioning the future, moral decision making, and prospection (Buckner and others 2008; Spreng and others 2009). Given this high similarity, it might be more informative to think of the AG beyond the boundaries of each domain. This short paragraph thus aims to provide a unified picture of AG function that transcends specific domains or tasks. For instance, as argued by Binder and others (2009), the AG may play a particular role in all tasks requiring fluent conceptual combination, such as sentence comprehension, discourse, problem solving, and planning (Binder and others 2009). In another model, the AG was proposed as a module of an *analyzing* block responsible for accessing subparts of stored items (Shalom and Poeppel 2008). But before discussing specifically the fundamental features that may define AG function, it is useful to see how the AG was portrayed in some previous seminal studies.

By thoroughly reviewing earlier neuropsychological and comparative studies, Geschwind (1965) argued that intermodal associations become powerful in humans thanks in part to the emergence of the AG, which acts as a “visual memory centre for words” and has contributed tremendously to the development of language and speech. Thirty years ago, Joseph (1982) defined the AG with Broca and Wernicke areas as components of a *language axis* that, with its complex interactions with the thalamic system, enables the formulation of speech and thought. Specifically, he proposed that the AG is “involved in the assimilation of diverse information variables, their integration, the calling-up of relevant associations, and functions as a necessary intermediary for all conscious functioning, particularly in the development and comprehension of language and thought. . . . [The AG] increases the capacity for the organization, categorization, and labeling of sensory-motor events” (Joseph 1982, p. 22). He then defined the AG as a processing center “where cross-modal associations such as visual, somasthetic and other sensory-motor concommitants are aroused, integrated, organized, assimilated, and finally comprehended” (Joseph 1982, p. 24). In addition, in his generalized model of sensation-to-cognition, Mesulam (1998) suggested that transmodal areas that include the posterior parietal cortex “provide critical gateways for transforming perception into recognition, word-forms into meaning, scenes and events into experiences, and spatial locations into targets for exploration.” In particular, thanks to its role in multimodal associations, the posterior parietal cortex (including the AG) also supports spatial awareness and working memory–executive function (Mesulam 1998).

Furthermore, Damasio (1989) introduced the concept of a *convergence zone* to describe the function of the posterior parietal cortex (including the AG) and other regions at the system level. Convergence zones are assumed to be amodal, and they sustain integration in a multimodal system (Damasio 1989). They are purposely considered “a critical gateway for accessing, binding and integrating information related to the conceptual representation and exploration of the extrapersonal space. . . . They register combinations of components in terms of coincidence or sequence, in space and time” (Damasio 1989). This framework has recently been adapted by Binder and Desai (2011), who proposed that the AG belongs to the convergence zones that store increasingly abstract representations of entity and event knowledge. They pointed out that the level of activation in the AG reflects the amount of semantic information that can be successfully retrieved from a given input, which suggests that the AG may play a unique role in the representation of event concepts (Binder and Desai 2011).

Fortunately, these models seem to agree on some fundamental features that shape AG function. These features include cross-modal associations (or, in Damasio’s model, *trimodal combinations*), integration, meaning, and event representations. Given also the consistent involvement of the AG in the default network, memory retrieval, and spatial and social cognition, it is important to reckon other extra features that include the sense of agency and action awareness (Farrer and others 2008; Farrer and Frith 2002; Kim 2010; Sperduti and others 2011). These extra features of agency and action awareness complete the set of key features that embody the multiple roles of the AG because both seem necessary to accurately compass the *dynamic* nature of semantics (i.e., as events and experiences) where persons, concepts, objects, and actions bind in time and space (Zhuge 2010).

To conclude this paragraph, it becomes clear that the AG resembles a “core facility” used by different subsystems to access concepts when interfacing perception-to-recognition-to-action. More specifically, given its rich connectivity and its location where multisensory information converges, the AG resembles a cross-modal integrative hub that gives sense and meaning to an event within a contextualized environment, based on prior expectations and knowledge, and toward an intended action. Although *integration* and *amodality* have been associated with more anterior temporal regions (see reviews in Jung-Beeman 2005; Patterson and others 2007; Stowe and others 2005), it is plausible that the AG supports initial (or first-order) integration that provides direct access to conceptual representations. This is supported by recent evidence that showed, for instance, the involvement of the AG in audiovisual speech integration (Bernstein and others 2008) and face-voice integration during person recognition (Joassin and others 2011). However, this does not preclude strong interactivity between the AG and other integrative hubs (Patterson and others 2007) that may increase with task demands (e.g., Obleser and others 2007).

### A Unified Account of AG Multiple Functions

**Figure 3.**
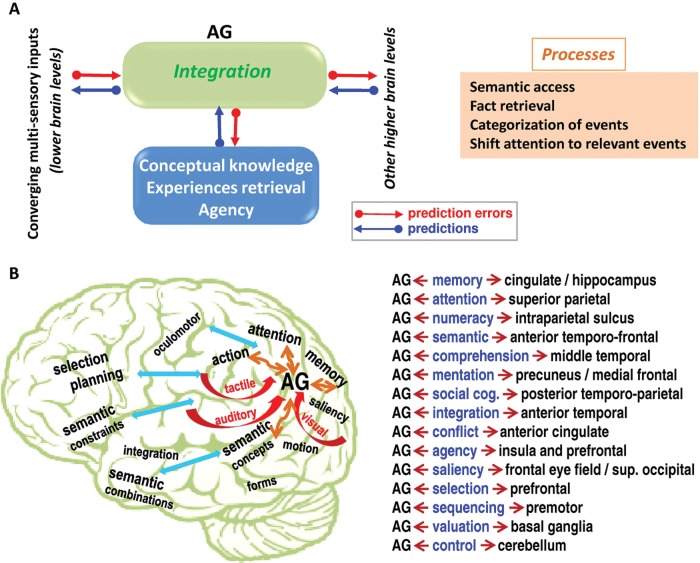
(*A*) Provides a unified framework that could account for the multiple functions of the angular gyrus (AG). Converging multisensory inputs are integrated in the AG (green box) in a context-dependent fashion. Top-down predictions (blue box) shape the integration of the converging inputs, and these predictions are generated on the basis of prior knowledge about the external world, similar learned experiences that can be retrieved, and the attribution of own action (i.e., the sense of agency). Other top-down predictions may come from other subsystems that code intention, saliency, and priority given to specific targets or events of interest. The integration in the AG proceeds via the recurrent exchange of bottom-up prediction errors (red arrows) and top-down predictions (blue arrows) until prediction error is minimized in the sense of the predictive coding framework (Friston 2010). This integration ultimately contributes in comprehending and reasoning about external events or internal mental representations and results in a set of core processes (orange box) that include events categorization, semantic access, fact retrieval, and shifting attention to relevant information. (*B*) Schematically illustrates the complex interplay between the AG and other distributed subsystems. It shows the convergence of different multimodel inputs to the AG (red arrows) and the interactions with different subsystems (orange arrows) that include memory, attention, action, and semantics. Interactions with other potential subsystems are indicated with blue arrows. The definition of semantic combinations and constraints is based on Price’s (2010) review, and the role of anterior temporal regions in semantic integration is based on Jung-Beeman’s (2005) review. Candidate regions that may strongly interact with the AG are listed with the most likely function/domain of interest. This is an oversimplified illustration because each domain/system contains several regions that can differently interact with the AG.

Figure 3 schematically illustrates a unified framework that could account for the different processes/domains that activate the AG as detailed above. This framework is borrowed from the popular predictive coding framework (as reviewed in Friston 2010) that models the brain as a hierarchical inference engine that is trying to optimize probabilistic representations of what caused its sensory input. Specifically, Figure 3A assumes the AG as an interface between the converging bottom-up multisensory inputs and the top-down predictions. Top-down predictions are conveyed by backward connections and are compared with the representations being generated at the AG, with the difference between the two reflecting the prediction error. This prediction error is then forwarded to higher levels to adjust and optimize the predictions. The recurrent exchange of bottom-up prediction errors and top-down predictions proceeds until prediction error is minimized at all levels of the system. Thus, the cross-modal integration in the AG can conceptually be seen as the sum of such recurrent exchange that happens at the level of the AG. The top-down predictions are based on previous knowledge of the external world, similar learned experiences that can be retrieved, and the awareness of own action (sense of agency). They may also come from other subsystems that maintain the intention (i.e., the planned action/decision to be made) and the saliency and the priority given to particular events of interest. The core processes that result from such integration within the AG translate into the categorization of events, access to semantics, fact retrieval, and shifting attention toward relevant information.

This framework can explain the multiple functions that implicate the AG. For instance, access to semantics is a key process in language comprehension and sentence reading. Likewise, fact retrieval reflects the retrieval of learned rules and facts that are important in number processing and in print-to-sound conversion during reading. Categorization of events and shifting attention to relevant information are important in social cognition, memory, and spatial cognition. In the case of the default network, the manipulation of conceptual knowledge, the sense of agency, and the retrieval of previous experiences (as predictions in Figure 3A) can modulate AG activity even in the absence of external sensory inputs. These processes are likely to have a hemispheric bias favoring more the left than the right hemisphere or vice versa; for instance, attention might lateralize toward the right AG and semantic access might lateralize toward the left AG. Critically, it is important to keep in mind that the exact role of the AG depends on the set of regions it is interacting with during a given task/process, as illustrated in Figure 3B. All these issues warrant further investigations.

## Multiple Subdivisions

In the section above, the AG was assumed so far as one single region with homogeneous anatomical and functional properties, thus ignoring the large variability across studies in the localization of AG activations. For instance, in a previous meta-analysis of semantic processing, Binder and others (2009) showed a wide distribution of activated peaks across 120 functional studies in bilateral AG (see Figure 2 in Binder and others 2009). Consistent AG peaks across multiple reviews have been considerably variable, as illustrated here in Table 1. This large variability in the AG may potentially reflect the existence of multiple subdivisions within the AG. Indeed, an increasing literature has defined the AG as an aggregate of multiple subdivisions where each subdivision can be characterized by specific functional and connectivity patterns (e.g., Andrews-Hanna and others 2010; Bahnemann and others 2010; Brownsett and Wise 2010; Caspers and others 2008; Eidelberg and Galaburda 1984; Kim 2010; Mars and others 2011; Naidich and others 1995; Nelson and others 2010; Nickel and Seitz 2005; Price GR and Ansari 2011; Rushworth and others 2006; Seghier and others 2010; Seghier and Price 2009; Sharp and others 2010; Uddin and others 2010; Vandenberghe and Gillebert 2009; Yeo and others 2011). This literature has provided an interesting framework for reporting and interpreting AG activations with greater definition, where each subdivision is allowed to have distinct contributions in a given task. This literature is briefly presented below with emphasis on anatomical, connectivity, or functional parcellation.

### Anatomy-Based Parcellation

Previous neuroimaging studies have suggested a possible anatomical segregation of the AG according to the anatomical variability in the sulci and gyri around the posterior inferior parietal region. For instance, following a detailed anatomical analysis of the variability in the horseshoe shape of the angular gyrus, it was suggested that an additional accessory pre-angular (pre-AG) area can be reliably defined at the anterior part of the angular sulcus (Naidich and others 1995). In addition, Eidelberg and Galaburda (1984) suggested three possible subareas in the AG using cytoarchitectonic parcellation on 8 postmortem brains (Eidelberg and Galaburda 1984). Recently, using an observer-independent cytoarchitectonic analysis on 10 postmortem brains, two cytoarchitectonic subdivisions were traced and labeled as areas PGa and PGp with high consistency across subjects (Caspers and others 2008; Caspers and others 2006) (see Fig. 1C). This recent anatomical parcellation into PGa and PGp is now widely used in both connectivity and functional region-based analysis.

### Connectivity-Based Parcellation

Using diffusion tensor imaging, different structural connectivity patterns were observed at different parts of the AG. For instance, tractography-based parcellation of the inferior parietal lobule identified five clusters that included two subdivisions within the AG (Mars and others 2011): a first cluster in the posterior-ventral part of the AG and a second cluster in the anterior-dorsal part of the AG. These two clusters resembled, respectively, the cytoarchitectonic subdivisions PGp and PGa shown in Figure 1C. Using resting-state functional connectivity analysis, it has been suggested that the posterior-ventral subdivision of the AG connects to the parahippocampal gyrus, whereas the anterior-dorsal subdivision of the AG connects more strongly with the anterior prefrontal cortex (Mars and others 2011). Similarly, a direct comparison between the resting-state functional connectivity patterns of seed regions PGa and PGp revealed greater connectivity for PGa with the caudate, bilateral frontal poles, and posterior and anterior cingulate, as well as greater connectivity for PGp with the hippocampus, parahippocampal gyrus, medial prefrontal cortex, and precuneus (Uddin and others 2010). Although tractography from both subdivisions PGp and PGa showed strong connectivity with the caudate, there was stronger structural connectivity from PGp than PGa with the hippocampus and the parahippocampal gyrus (Uddin and others 2010).

### Function-Based Parcellation

Some recent functional neuroimaging studies have reported different contributions of distinct parts of the AG over variable tasks and processes. This was, in particular, shown in the left AG. For instance, Nelson and others (2010) used a combined analysis on the profile of memory retrieval–related activity and the membership to large-scale brain networks to dissociate six subregions in the lateral parietal cortex. Two subdivisions were located near the AG: a ventral region where activity correlated with that in the medial prefrontal cortex and a dorsal region where activity strongly correlated with that in the superior frontal gyrus (Nelson and others 2010). Using passive perception of letters and Arabic digits, the left AG was also segregated into a dorsal subdivision that was activated by both familiar letters and digits and a ventral subdivision that was strongly activated for digits compared with letters (Price GR and Ansari 2011).

During speech comprehension, two AG clusters were segregated by manipulating the semantic and acoustic difficulty in a task that required making decisions based on the semantic relatedness between heard nouns (Sharp and others 2010). A first ventral posterior AG cluster showed stronger activation for high semantic versus high acoustic difficult conditions, whereas a dorsal anterior AG cluster showed stronger activation for high semantic versus control speech (Sharp and others 2010). Using similar semantic decision tasks but on written words and pictures of familiar objects, Seghier and others (2010) showed a reliable intersection between the semantic network and the default network at a precise AG location that served as a functional landmark to dissociate three subdivisions in the left AG (Seghier and others 2010). A first subdivision, located at the site of the overlap between the two networks, is involved in semantic associations regardless of the presence or absence of a stimulus; dorsal to the overlap is a second subdivision involved in searching for semantics in all visual stimuli; and ventral to the overlap is a third subdivision involved in the conceptual identification of visual inputs (Seghier and others 2010). On the other hand, evidence for similar subdivisions in the right AG is scarce, although one study of number processing dissociated two ventral and dorsal clusters in the right AG that varied in activation level with number selectivity, response time-related effects, and stimulus-independent task effects (Cappelletti and others 2010).

**Figure 4.**
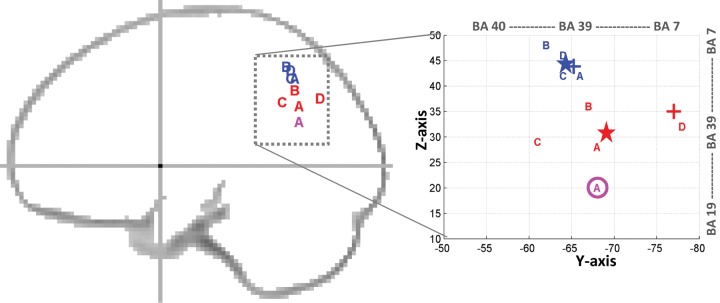
Projection of Montreal Neurological Institute (MNI) coordinates of previous angular gyrus (AG) subdivisions on a schematic sagittal outline (*left*, at *x* = −30 mm) and a zoom centered at the AG (*right*). A = Seghier and others (2010), B = Nelson and others (2010), C = Price GR and Ansari (2011), and D = Sharp and others (2010). Dorsal coordinates are shown in blue and ventral coordinates are shown in red. A third more ventral subdivision at *z* = +20 mm is shown in magenta (according to Seghier and others 2010). Average locations are illustrated with a star (blue for dorsal subdivision at [*x* = −35, *y* = −64, *z* = 45], red for ventral subdivision at [*x* = −42, *y* = −69, *z* = 31]). The average locations here are remarkably similar to the two subdivisions used in a recent seed-based resting-state functional connectivity study over a large sample of 1000 subjects (noted as PGpd and PGpv in Table 4 and Figure 30 of Yeo and others 2011). Approximate location of the center of gravity of cytoarchitectonic areas PGa and PGp (Caspers and others 2008) is indicated by “+” in blue and red, respectively. BA = Brodmann area.

Figure 4 illustrates the projection of the coordinates of the different left AG subdivisions identified in the four functional neuroimaging studies cited above (cf. Nelson and others 2010; Price GR and Ansari 2011; Seghier and others 2010; Sharp and others 2010), as reported in the standard Montreal Neurological Institute (MNI) space. By grouping these MNI coordinates and looking for consistent localizations, two AG subdivisions can clearly be identified (Fig. 4): 1) a first subdivision that is located dorsally in the AG with mean coordinates at [*x* = −35, *y* = −64, *z* = 45] and 2) a second subdivision located ventrally in the AG with mean coordinates at [*x* = −42, *y* = −69, *z* = 31]. More specifically, dorsal AG subdivision is likely to participate in “bottom-up” processes during semantic search (Price CJ 2010; Seghier and others 2010), fact retrieval (e.g., Ischebeck and others 2009), and the (automatic) allocation of attention to memory (Cabeza and others 2008; Ciaramelli and others 2008), whereas ventral AG subdivision may exert a “top-down” influence in self-referential processing (Kim 2010), providing semantic constraints (Price CJ 2010; Seghier and others 2010), and making judgments on mental representations (e.g., Bahnemann and others 2010; Mar 2011). The dorsal subdivision is bounded by the lateral bank of the intraparietal sulcus, and the ventral subdivision most likely overlaps with the deactivated regions of the default network. It is worth noting that a third subdivision, located more ventrally at MNI–*z* coordinates of *z* = +20 mm, was also identified by Seghier and others (2010), as illustrated in magenta in Figure 4. This third ventral AG subdivision showed stronger semantic responses when stimuli were pictures than written words, suggesting its role in direct access to concepts from visual inputs (Seghier and others 2010). Similarly, during social cognition tasks, Bahnemann and others (2010) observed a strong overlap between theory-of-mind tasks and moral judgment tasks at this more ventral AG subdivision that was distinct from other dorsal AG responses.

To conclude this section, both anatomical and functional evidence supports the existence of a high-definition map in the AG. This is particularly visible along the ventral-to-dorsal axis in the left AG, as illustrated in Figure 4. Future studies can use this spatial parcellation of the AG as a roadmap to report their activations.

## Future Work

The studies reviewed here have provided valuable insights to our understanding of the exact contribution of the AG in cognition. Several other issues warrant further investigations, as briefly listed below:

### Identify the set of core regions that interact with the AG in different processes and how these interactions are modulated by task demands (as illustrated in Fig. 3B)

This important issue would benefit from the popularization of effective connectivity techniques that allow the direction and strength of interregional coupling to be estimated. For instance, Carreiras and others (2009) demonstrated a “top-down” role of the AG on posterior occipital areas during reading aloud relative to object naming (Carreiras and others 2009). Similar studies are thus needed to depict a mechanistic account for AG role(s). A particularly interesting question is the nature of interactions that the AG carries with the rest of the semantic network. The semantic system is composed of a large set of nodes (Binder and others 2009) that may play different roles in semantic processes, including the pars orbitalis, the middle temporal gyrus, and the temporal pole (for more details, see Price CJ 2010). Moreover, there is a lack of literature regarding the potential interactions between the AG and cerebellar regions, particularly when considering the contribution of the cerebellum to different cognitive processes (Schmahmann 2010). For instance, a recent resting-state connectivity analysis has revealed strong functional connectivity between the posterior parietal cortex (including the AG) and a supramodale zone of the cerebellum (see O’Reilly and others 2010).

### Visualize the dynamics of AG activation using high-temporal resolution techniques

In this brief review, it was not possible to do justice to previous electroencephalogram, transcranial magnetic stimulation, or magnetoencephalogram studies because this literature requires its own review. For example, these techniques can help to reveal whether AG activation happens at earlier or later latency than frontal and temporal regions and whether this latency changes with task demands and modality.

### Characterize lateralization in the AG and how it is modulated by task and modality

This issue relates to the possible differences in functional properties in the left and right AG over varieties of tasks (Jung-Beeman 2005; Lindell 2006). For instance, as shown above, the involvement of the AG for semantics, spatial cognition, or number processing may vary between left and right hemispheres. A systematic analysis of lateralization effects in the AG will provide important clues for future models of AG function. In the same way, the different AG subdivisions shown above are mainly identified in the left hemisphere. Future studies are needed to investigate whether the same functional subdivisions exist in the right AG.

### Characterize specific deficits associated with AG damage

It has been shown that damage to the AG has consequences on a range of skills, including speech comprehension, finger agnosia, spatial disorientation, acalculia, agraphia, and dementia (e.g., Ardila and others 2000; Corbett and others 2009). The wide range of deficits speaks volume to the multiple tasks and processes that depend on the integrity of the AG. Future work can report AG damage at high definition, as reviewed here, to identify whether these variable deficits may reflect damage to specific subparts of the AG.

### Explore interindividual variability in AG function

There is an increasing interest in characterizing variability in function between subjects because it can reveal the different cognitive strategies used by subjects when performing the same task (Miller and others 2012; Seghier and Price 2009). Developmental factors may also contribute to such variability, and thus studies are needed to test whether AG structure and function vary over the life span. For instance, it has been shown that the AG is one of the few brain regions where structural asymmetry decreases with age (Kovalev and others 2003). In the same context, the impact of other demographic (gender and handedness) and genetic variables on the anatomy and function of the AG warrants further studies.

### Compare the size, location, and connectivity of the AG across different species

For instance, it has been shown that a major temporal lobe projection of the arcuate fasciculus in humans is smaller or even absent in chimpanzees and macaques (Rilling and others 2008). This topic warrants systematic investigation because it can provide new clues to explain the disproportionate expansion of the multimodal associative regions in humans and their significant contribution to our cognitive and linguistic abilities (see discussion in Rosa and Tweedale 2005; Sherwood and others 2008).

## Conclusion

This brief review aimed to bring together previous findings to construct a unified picture of the AG during all processes, from perception to action. It highlights the integrative role of the AG in comprehension and reasoning—for instance, when manipulating conceptual knowledge, reorienting the attentional system toward relevant information, retrieving facts for problem solving, and giving meaning to external events based on stored memories and prior experiences. This review also discussed the spatial fractation of the AG into multiple subdivisions that may contribute in “bottom-up” and “top-down” mechanisms across numerous tasks. Future studies have to clarify how the AG communicates with other subsystems to continuously give meaning and sense to the external world. Finally, this highly selective review recognizes that cracking the code that uniquely defines AG function is still an ongoing endeavor.
